# Therapeutic approaches targeting Apolipoprotein E function in Alzheimer’s disease

**DOI:** 10.1186/s13024-020-0358-9

**Published:** 2020-01-31

**Authors:** Tosha Williams, David R. Borchelt, Paramita Chakrabarty

**Affiliations:** 1 Center for Translational Research in Neurodegenerative Disease, University of Florida, Gainesville, FL 32610 USA; 2 Department of Neuroscience, University of Florida, Gainesville, FL 32610 USA; 3 McKnight Brain Institute, University of Florida, Gainesville, FL 32610 USA

**Keywords:** Apolipoprotein E, Tau, Amyloid β, Therapy, Alzheimer’s disease

## Abstract

One of the primary genetic risk factors for Alzheimer’s disease (AD) is the presence of the Ɛ4 allele of apolipoprotein E (APOE). APOE is a polymorphic lipoprotein that is a major cholesterol carrier in the brain. It is also involved in various cellular functions such as neuronal signaling, neuroinflammation and glucose metabolism. Humans predominantly possess three different allelic variants of *APOE*, termed E2, E3, and E4, with the E3 allele being the most common. The presence of the E4 allele is associated with increased risk of AD whereas E2 reduces the risk. To understand the molecular mechanisms that underlie *APOE*-related genetic risk, considerable effort has been devoted towards developing cellular and animal models. Data from these models indicate that *APOE*4 exacerbates amyloid β plaque burden in a dose-dependent manner. and may also enhance tau pathogenesis in an isoform-dependent manner. Other studies have suggested APOE4 increases the risk of AD by mechanisms that are distinct from modulation of Aβ or tau pathology. Further, whether plasma APOE, by influencing systemic metabolic pathways, can also possibly alter CNS function indirectly is not complete;y understood. Collectively, the available studies suggest that APOE may impact multiple signaling pathways and thus investigators have sought therapeutics that would disrupt pathological functions of APOE while preserving or enhancing beneficial functions. This review will highlight some of the therapeutic strategies that are currently being pursued to target APOE4 towards preventing or treating AD and we will discuss additional strategies that holds promise for the future.

## Background

### Apolipoprotein E4 is the major genetic risk factor in Alzheimer’s disease

Alzheimer’s disease (AD), neuropathologically characterized by extracellular amyloid β (Aβ) deposition and intracellular neurofibrillary tangles (NFT) of tau protein, is the most prevalent neurodegenerative dementia affecting millions of people worldwide [1]. One of the primary genetic risk factors for sporadic AD, also referred to as late onset AD (LOAD), is the presence of the E4 isoform of apolipoprotein E (APOE) protein [2].

Humans have three major *APOE* alleles (E2, E3, and E4) [3]. *APOE*3 is the reference allele present in the majority of the population; the *APOE*4 allele increases the risk of AD in a dose- and age-dependent manner whereas the *APOE*2 allele is associated with reduced risk of AD [4–6]. Though there are variations based on sex and ethnicity, it is estimated that *APOE*2 homozygotes have a 40% reduced risk of developing AD [7]. Presence of *APOE2* delays the age of onset in the Paisa kindred of familial AD cases [8], reinforcing the idea that APOE2 isoforms are protective against familial AD. In humans, the *APOE2* allele while being protective against AD, is associated with elevated plasma levels of cholesterol and triglycerides and a condition called dysbetalipoproteinemia that is associated with coronary artery disease [9]. On the other hand, *APOE4* is associated with increased risk of atherosclerosis and increasing risk of AD by as much as 8-12x in homozygotic humans. There is a general consensus in the literature that AD patients with the APOE4 isoform have accelerated onset of dementia, worse memory performance and higher Aβ burden than APOE4 non-carriers [10], though the isoform-dependent effects on tauopathy remain unclear [11, 12]. APOE4 can also exacerbate functional abnormalities such as neuronal network connectivity independent of gross structural changes or AD type proteinopathy [13]. These topics have been covered previously in excellent reviews and therefore not discussed further [10, 14, 15]. These data suggest that it may be necessary to both restore some critical APOE function in E4 carriers while also inhibiting the activity of APOE4 in promoting AD-related Aβ proteinopathy [14].

### Peripheral and CNS pools of APOE are independent

APOE is a 299 amino acid protein, with an apparent molecular mass of ~36kDa whose primary function is that of a cholesterol transporter [14]. The three isoforms differ by one amino acid each at positions 112 and 158 that has profound effects on their functions. Both APOE2 (Cys112, Cys158) and APOE3 (Cys112, Arg158) preferentially interact with small, phospholipid-enriched high-density lipoproteins (HDL), while APOE4 (Arg112, Arg158) has higher propensity to be associated with larger, triglyceride-enriched lipoproteins or VLDL [16]. A further distinction is that among all the isoforms, APOE2 has the lowest binding affinity for low-density lipoprotein (LDL) receptors [17]. Mice have a single allele of Apoe that differs at multiple positions from human APOE, but encodes Arg at the positions cognate to 112 and 158 of human APOE. Most of what is known about APOE has been derived from studies in mice and human cell culture models. Studies in mice have examined both endogenous mouse Apoe and expressed human APOE. For the purposes of this review, we will use the human and mouse nomenclature interchangeably as appropriate for the model systems used, defaulting to APOE when discussing general features of APOE biology.

In the CNS, APOE is primarily synthesized by astrocytes and in certain circumstances, it is also produced by microglia and neurons [18–21]. APOE has myriad functions in the CNS that include immunomodulation, signal transduction, proteostasis regulation and synaptic plasticity [14, 22]. The peripheral pool of plasma APOE is produced mainly in the liver, and to a lesser extent by the adrenal gland and macrophages. In the periphery, in addition to regulating lipid metabolism, APOE has a key role in controlling cardiovascular function and systemic inflammation [23]. This pool of APOE exists mostly independent of the CNS pool under normal circumstances [24, 25]. An important difference between the CNS and peripheral APOE pools is that only peripheral APOE4 shows faster turnover rate compared to APOE3 and APOE2 in humans and humanized mice [22, 23]. Astrocytic and plasma APOE lipoprotein particles are also structurally different and the former is thought to lack the cholesteryl ester core [26]. Therefore, it is possible that the structure-function relationship of peripheral and CNS pools of APOE to the development of AD and non-AD pathologies might be distinct, suggesting that these two pools of APOE can potentially act independently as risk factors in regulating pathogenesis during normal aging or in neurodegenerative dementias.

Because of the pleiotropic functions of APOE isoforms in the CNS and periphery, mechanistically dissecting the role of APOE in the context of AD and related disorders is fraught with complications. This uncertainty over potential mechanism of action creates a conundrum in that the E4 allele may cause disease by both a loss of function or gain of function, depending on the cellular context [14]. A recent report had serendipitously identified a mouse model with intact peripheral Apoe levels and thus normal plasma lipid profile but with extremely low levels of brain Apoe. These mice have impaired synaptic plasticity but their spatial memory skills are intact [24], suggesting that peripheral and CNS APOE may have distinct effects on CNS function. On the other hand, absence of hepatic APOE does not affect the APOE4-dependent induction of Aβ pathologies in young *APP*/*PS1* female mice, suggesting that plasma APOE4 may have little influence on initiation of Aβ pathologies in the brain [27]. With this knowledge, it is reasonable to explore treatment options that would preferentially modify the CNS pool of APOE without affecting the peripheral sources, thus also avoiding systemic metabolic syndromes.

### Rodent models as exemplars of human APOE function

The *Apoe* deficient mice, *Apoe* hypomorphic mice and *APOE* knock-in mice have been key resources in the field of atherosclerosis biology, cardiovascular disease and peripheral inflammation [28]. For the most part, the data are concordant between mouse studies and humans [29]. However, there are some critical differences between mouse and human lipoprotein biology that can impact the interpretation of APOE-related studies in mice. In mice, circulating cholesterol is predominantly associated with HDL whereas it is bound to LDL in humans [30]. In addition, mice lack the cholesteryl ester transfer protein (*CETP*) gene which transfers cholesteryl esters and triglycerides between lipoproteins [31].

Perhaps the most commonly used models to study human APOE function in the CNS are the human *APOE* targeted replacement (TR) mice from Nobuyo Maeda’s lab [32–34]. The *APOE4* TR mice, in which the endogenous *Apoe* gene has been replaced with human *APOE4*, display various phenotypes including altered cholesterol trafficking in the brain, blood brain barrier (BBB) leakiness and cognitive deficits [35–39]. However, simply replacing the endogenous mouse *Apoe* gene with the human *APOE4* gene does not produce the primary neuropathologies (Aβ and NFT) found in AD patients. Overall, the lack of spontaneously occurring AD-type pathology in *APOE4* TR mice has limited its use as a stand-alone model of AD.

### Concurrence of experimental data across different systems

**Fig. 1:**
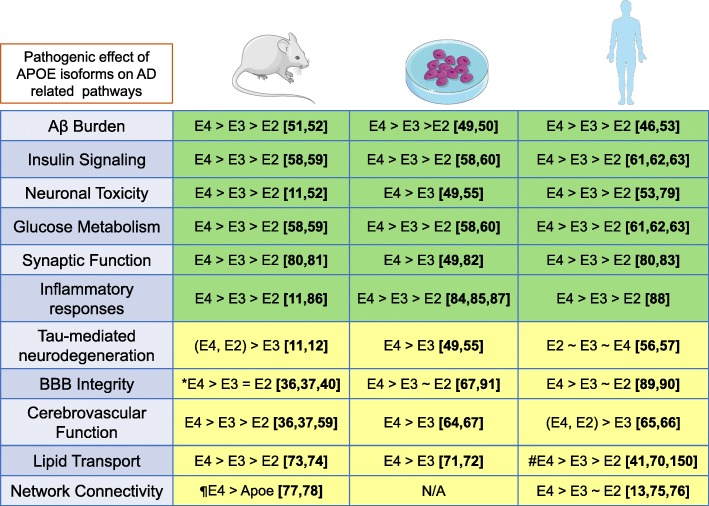
Congruence of the effects of apoE between human studies, mouse models of AD and in vitro cell culture models. apoE influences multiple pathways in the AD cascade in an isoform-dependent manner. We compared the concurrence of available research data in mouse models and in vitro models versus clinical studies with human patients. Pathways indicated in the green color indicate a broad consensus of APOE isoform effect between mice, men and in vitro models where E4 is associated with an increased pathological risk when compared to E3 or E2 isoforms (E4>E3>E2). Data from the pathways indicated in yellow background are not in complete congruence between human studies, mouse model experiments and in vitro data. Interestingly, even within a set of studies in a given experimental system, there is disagreement in between the observations, which is marked by superscripted symbols that refers to the disparate studies. The symbols (< or >) indicate the order of increased pathological effect for the APOE isoforms. The effects listed here are specific to only classical AD pathology and excludes data on α-synuclein and TDP43 which are associated with diseases such as PDD and DLB. *, conflicting reports [see ref 40]; #, conflicting reports [see ref 41]; ¶, studies compared *APOE4 TR*, *Apoe* KO, and wild type C57BL6J mice. The references presented are representative and not an exhaustive list

A large number of studies have used rodent models (such as *APOE TR*), in vitro models including human induced pluripotent stem (iPS) cells and primary rodent cultures as well as data from human biosamples to delineate apoE-related pathologies. Most of the studies show isotype-specific and directionality-specific concordance between these experimental paradigms (Fig. 1). For example, the isoform-dependent effects of APOE on Aβ clearance and Aβ aggregation are in complete agreement in these different systems [42–53]. As in humans, presence of APOE4 increases Aβ deposition burden in *APP* transgenic mice relative to age-matched *APOE2 TR* mice. This has also been demonstrated in human iPSC-derived glial cultures, where APOE4 impairs glial Aβ uptake and phagocytosis compared to APOE3 [48]. On the other hand, there is a current lack of consensus regarding the relationship between tauopathy and APOE isoform as demonstrated by studies showing a pathogenic interaction of tau to APOE4 [11, 54] or APOE2 [12]. Further, human iPSC derived neurons [55] as well as organoids [49] that express APOE4 accumulate higher levels of phosphorylated tau when compared to neurons expressing APOE3. Importantly, data from humans present no clear association between *APOE4* genotype and severity of NFT pathology [56, 57]. Different APOE isoforms have differential pathogenic effect on various metabolic pathways such as cardiovascular function, lipid transport, insulin signaling and glucose metabolism across these model systems [36, 37, 58–67]. There is a clear consensus regarding APOE4 isoform-dependent pathogenic effect on cardiovascular function in mouse models, in vitro studies and human studies [34, 35, 41, 68]. In the case of lipid transport, several studies have shown that *APOE4* carriers have increased hypolipidated APOE compared to APOE3 and APOE2 carriers [69] along with reduced APOE levels in the CSF of Aβ-positive *APOE4* carriers [70]. These observations hold true in primary rat neuron cultures and human iPS cell-derived astrocytes [71, 72] as well as *APOE TR* mice [73, 74]. However, there are some conflicting reports from human studies, which did not observe any isoform-dependent differences in APOE levels in the CSF collected from individuals at different ages [41].

APOE has been implicated in other metabolic and cognitive functions. In the case of insulin signaling and glucose metabolism, data from human brain scans, mice and in vitro cell culture largely agree that APOE4 isoforms cause the most functional impairment [58–63]. In terms of regulating brain function, APOE4 is the most pathogenic in terms of brain connectivity and default mode network function in humans whereas the evidence comparing network connectivity in mouse models carrying *APOE4* genotype is uncertain as the experimental controls did not include the *APOE3* or *APOE2* mice cohorts [13, 49, 75–83]. In human iPS derived neurons, APOE4 led to elevated number of synapses and increased frequency of synaptic transmission [49]. Regarding inflammation, there is a general agreement across models that APOE4 is the most pathogenic [11, 84–88]. In contrast, there is evidence implicating APOE4 in impaired BBB integrity in humans, mouse models and cell culture models, although a study in *APOE4 TR* mice did not reveal any APOE-related dysfunction in BBB [36, 37, 40, 67, 89–91]. Overall, most of the data from rodent models and human patients show congruence (Fig.1). However, given that there are basic differences between mice and human lipid profiles as well as the structure of apoE itself, it is important to remain cautious of the inherent variations that might affect directly translating APOE targeted therapeutics from mouse models to humans.

### Therapies in AD

AD still has no effective treatments or therapies despite years of research. Dozens of drugs have proceeded to clinical trials, ranging from Aβ targeting antibodies to therapies targeting tau or metabolic pathways [92]. Several factors may have influenced these discouraging outcomes - perhaps the drugs are targeting the wrong pathological substrates, or that the treatments are being administered too late, or that a multi-target drug design is needed [93]. With the steady growth of an aging population, the increasing cost for care, and the failure of therapies in clinic, there is a call for more targeted ‘precision therapy’ - treating AD patients stratified based on their *APOE* genotype.

It is well-known that targeting anti Aβ immunotherapies to patients stratified for *APOE* genotype can lead to better outcomes. In particular, in MCI patients the *APOE4* allele seems to adversely affect the therapy outcomes by modulating the treatment efficacy (disease progression) or safety profile (vulnerability to brain edema) relative to other *APOE* alleles [94]. Having said that, AD therapies directly targeting specific APOE isoforms are still mostly in developmental phases [95]. It is also possible that such APOE targeted therapies could help with co-morbidities associated with dementia or aging, such as diabetes and cardiovascular disease for E4 carriers, vascular dementia for E4 carriers, neuroinflammation for E4 carriers and type III hyperlipoproteinemia for E2 carriers. Whether APOE by itself is druggable is debatable; however, it is tempting to suggest that targeting CNS APOE specifically early in the disease process could alter the pathologic trajectory of AD either directly by altering CNS pathologies like Aβ and tau and perhaps indirectly by influencing related sequelae, such as inflammation, metabolic impairment and vascular dysfunction. Ultimately, it is possible that a cocktail of drugs targeting APOE function in conjunction with other anti-Aβ approaches that either limit Aβ plaques or inhibit Aβ production can be used at different stages of the disease to achieve significant disease modification.

## APOE as a therapeutic target in AD

In the next few sections, we will consider currently available preclinical interventions, therapies that are in early clinical studies in AD as well as some new research on emerging targets that target APOE specifically (Table 1).

**Table 1:** A selection of APOE based therapeutics used in rodent models and and clinical testing

Drug	Rationale	Developed by	Reference/Clinical Trial Identifier
CS-6253	Increase APOE lipidation by activating ABCA1	Tel Aviv University/Artery Therapeutics	*Ref* 125
CN-105	APOE mimetic	CereNova/AegisCN LLC	Phase1: NCT02670824 (ICH); *Ref* 231
Phthalazinones, pyrazolines	Small molecule structure-correctors	Gladstone Institute/E-Scape bio	*Ref* 132
APOE antibody	Targeting non-lipidated APOE	Washington University/Denali therapeutics	*Ref* 99
Anti-sense oligonucleotide	Reduce expression of APOE4	Washington University/Ionis	*Ref* 104
Gene Therapy	Biological: AAVrh.10 hAPOE2 vector	Cornell University	Phase 1: NCT03634007
Bexarotene	Alter APOE production, APOE lipidation and Aβ clearance	ReXceptor Inc. and C2N	Phase 1: NCT02061878 Outcome: No change in Aβ; increased CSF APOE
		Cleveland Clinic	Phase 2:NCT01782742 Outcome: No benefit in APOE4 patients; *Ref* 114
Probucol	Cholesterol lowering drug	McGill University/Douglas Hospital Research Center	Phase1/2: NCT02707458 *Ref* 232
AGB101	Reduce APOE4-dependent abnormal hippocampal network activity	Medical College of Wisconsin	Phase 2: NCT03461861 *Ref* 233
Rosiglitazone	Anti-diabetic (APOE allele dependent response)	GlaxoSmithKline	Phase3: NCT00348140 Outcome:No effect on mild to moderate AD; *Ref* 234
Epigallocatechin gallate (EGCG) + multimodal intervention (diet, exercise)	Correct APOE4-dependent cognitive decline	Parc de Salut Mar	Recruiting: NCT03978052. No direct references found but see *Ref* 235
Exercise	Relationship of APOE4 to CBF and blood-based biomarkers (IGF-1, VEGF, BDNF)	University of Kansas Medical Center	Recruiting: NCT04009629 *Ref* 236

### Altering levels of APOE4 as a potential disease modifying therapy

APOE, especially APOE4, binds to Aβ, playing a key role in Aβ deposition and clearance. Several studies have shown that simply reducing APOE4 levels (such as cre-mediated excision of APOE4 or creating haploinsufficient APOE4 models) lowers brain Aβ levels in *APP* transgenic mice [96, 97]. Other approaches such as blocking Aβ-APOE4 interaction can also lead to beneficial effects, prompting the development of strategies to either reduce the availability of APOE4 or prevent its toxic interactions.

#### Anti-APOE4 immunotherapies

Similar to anti-Aβ antibody-based therapies, the idea behind anti-APOE4 antibodies is that these antibodies will be able to cross the BBB and neutralize the negative effects of APOE4, even if only a small amount of antibodies can effectively enter the brain [98]. APOE4 has already been implicated in Aβ deposition, and along with other amyloid-associated proteins it is found in Aβ deposits. Thus, the idea is that if isoform-specific antibodies can sequester pathogenic forms of APOE, it can prevent Aβ build-up in the brain. Indeed, in mouse models, anti-APOE antibodies can efficiently inhibit the formation of Aβ deposits when introduced before the onset of pathology [98]. More interestingly, these antibodies were also able to attenuate plaque burden when introduced in mice with pre-existing Aβ deposits, suggesting that this antibody could work as a therapeutic agent [98]. In a subsequent study, anti-APOE antibodies also led to improved spatial learning performance and resting-state functional connectivity while having no effect on total plasma cholesterol in *APP* transgenic mice [99]. In this study, topical application of anti-APOE antibodies directly onto the brain prevented deposition of new Aβ plaques as well as cleared pre-existing plaques. The fact that these anti-APOE antibodies can disrupt the direct binding of apoE to Aβ deposits is very promising, as this might work synergistically with anti Aβ immunotherapy in *APOE4* patients to achieve a higher degree of Aβ reduction. More recently, Liao et al. reported that the antibody ‘HAE-4’ that preferentially recognizes the nonlipidated forms of APOE4/APOE3 over the lipidated versions is highly effective in preventing Aβ deposition by a FcγR-dependent mechanism in an *APP*/*APOE*4 mouse model [100]. Following direct infusion into the brain or following intraperitoneal administration, HAE-4 reduces total Aβ plaque burden but does not alter the fibrillar plaque load. Surprisingly, this antibody when administered peripherally was more efficient at CNS target engagement than when administered via direct brain infusion. This study is important in two ways – one that it demonstrates that non-lipidated forms of APOE4 may be preferentially pathogenic and second, that since the non-lipidated form of APOE4 is a small fraction of the total CNS APOE burden, this antibody would not be titered out by total APOE and could be efficacious at a lower or less frequent dose [101].

#### Antisense oligonucleotide therapy

Based on the hypothesis that the reduction of APOE4 expression could reduce Aβ accumulation and thereby alleviate Aβ pathology and cognitive deficits that typically follow, some groups have used antisense knockdown approaches. Antisense oligonucleotides (ASOs) are synthetic polymers that can be used as therapeutic agents by disrupting the synthesis of a particular protein and are considered as first line treatments in several neurodegenerative disorders such as polyneuropathy, muscular dystrophies and spinal muscular atrophy [102]. With regard to AD, there are only a few investigational ASO mediated therapies in clinical testing – a notable one being the anti tau ASO in Phase1/2 (BIIB080 from Ionis/Biogen/Washington University) [103]. In regard to targeting APOE expression with ASOs, the effort is still in the preclinical phases of testing.

ASOs targeting APOE receptors have also been tested in AD mouse models, for example an ASO specific for corrective splicing of ApoER2 resulted in improved synaptic function as well as learning and memory in the CRND8 mouse model of Aβ pathology [104]. In mouse models, ASO treatment targeting specifically APOE have been successful in reducing Aβ pathology in APP/PS1 mice when treated before the onset of Aβ deposition [105] (Table 1). However, it is unclear whether such knock-down strategies will work in the presence of pre-existing Aβ deposits, suggesting that this type of therapy may have a limited window of opportunity. On a positive note, compared to agonists of ApoE receptors that result in systemic adverse effects in lipid metabolism, ASOs do not show such side effects. Combined with the successful ASO based therapies being tested in Huntington’s and Amyotrophic Lateral Sclerosis (ALS) patients and the latest developments in ASO chemistry that can dramatically improve its pharmacokinetic and pharmacodynamic properties, ASO based therapies do have potential as promising future therapeutic for AD patients [106].

#### Upregulating APOE expression through nuclear receptor agonism

APOE expression is induced by the nuclear receptors, peroxisome proliferator-activated receptor gamma (PPARγ) and liver X receptors (LXR) in coordination with retinoid X receptors (RXRs) [107]. GW3965, an agonist for LXR, increases Abca1 and Apoe protein levels, reduces Aβ levels and improves cognition in the *APP*/*PS1* mouse model [108]. This result was Abca1-dependent, as GW3965 failed to alter Aβ levels in *APP* transgenic mice lacking *Abca*1. In another study, the RXR agonist Bexarotene (Targretin®), used to treat T cell lymphoma, was used in the *APP/PS1* transgenic mouse model. In a study involving a relatively small cohort, Bexarotene treatment reduced Aβ accumulation in an APOE-dependent manner when orally administered to these mice [109], though several groups were unable to recapitulate the beneficial effect on plaque burden in similar mouse models [110–112]. Based on the original study that showed that bexarotene was effective in both preventive and therapeutic modes, it was incorporated in a proof of mechanism Phase IB trial in E3/E3 healthy adults [113] (Table 1) as well as tested in a small cohort of AD patients, called BEAT-AD study [114]. In the BEAT-AD study, bexarotene lowered CNS Aβ levels (by PET imaging) but did not produce any cognitive benefits [114]. Unfortunately, bexarotene treatment increased blood lipid levels in these patients increasing their risk for stroke and heart attack. In a Phase IB proof of mechanism trial in young healthy *APOE3* carriers, researchers were able to measure plasma and CSF levels of APOE using the stable isotope leveling kinetics (SILK) method. Though APOE levels increased moderately in the CSF, there was no effect on synthesis or clearance of Aβ in CSF in these individuals [113]. One reason for this may be the poor CNS penetration of bexarotene in human patients (~low nM range) [113]. Notably, in mice the BBB is extremely permeable to bexarotene [115]. This finding raises a general cautionary issue regarding translation of drugs from rodents to humans. Coupled with the hepatotoxicity of bexarotene, the low CNS penetrance of drug resulted in disappointing forecast for translation to AD patients.

#### Stimulating APOE expression through HDAC inhibition

Histone deacetylase (HDAC) is a class of enzymes that remove acetyl groups from histones in DNA leading to gene silencing [116]. HDACs have been shown to play a central role in regulation of genes involved in the lipid metabolism pathway [117] as well as genes involved in long term memory formation and cognition [118]. A recent study in human astrocytoma cells showed that HDAC inhibition can stimulate APOE expression, independent of LXR and RXR [119]. Through the use of a phenotypic screening strategy utilizing various chemogenomics libraries, pan Class I HDAC inhibitors (MS275 and CI994) were found to increase APOE expression and secretion by astrocytes via an LXR-independent pathway [119]. These recent studies offer a new approach towards modulating APOE function.

### Restoring or recalibrating APOE functions can also alleviate CNS and peripheral pathologies

Another option for potentially exploiting APOE functionality for AD treatment is regulating or restoring the normal function of APOE that is typically lost, especially in patients carrying the *APOE4* isoform. Investigators have been pursuing strategies to raise overall levels of APOE function by increasing its lipidation as well as using small molecules to modulate APOE4 structure or function to more closely resemble APOE3. Some of these methodologies are primarily geared to recoup the loss of function in APOE in the *APOE4* patients whereas others target the toxic gain of function aspects that APOE4 may have on AD-related pathology.

#### Small molecules that enhance ABCA1-mediated APOE4 lipidation

Among all APOE isoforms, APOE4 is unique in that it has increased propensity of domain-domain interactions that reduces lipid binding to the C terminal domain leading to loss of stability and function [120, 121]. The presence of Arg112 in APOE4 enhances the intramolecular interaction between its N-terminal domain and the C-terminal domain via a salt bridge known as the APOE4 domain interaction. As a result, APOE4 is typically hypolipidated or ‘lipid-depleted’ which has been postulated to correlate with the pathogenicity inherent to APOE4 [69]. In general, APOE lipidation is highly reliant on the ATP-binding cassette transporter A1, or ABCA1, which moves lipids into apolipoproteins and is known to protect against atherosclerosis [122]. Indeed, humans lacking functional *ABCA1* have lower APOE levels and increased risk of AD and cardiovascular disease [123]. Consistent with this observation, deficiency of *Abca*1 exacerbates amyloidogenesis while overexpression of *ABCA*1 reduced the amyloid load in *PDAPP* transgenic mice [124]. Supporting the hypothesis that ABCA1-mediated lipidation is crucial Aβ clearance, subsequent studies have upregulated ABCA1 with peptides and various small molecules. An example of a small peptide that activates Abca1 is CS-6253 (Table 1). Intraperitoneal injection of CS-6253 into *APOE4* TR mice 1) upregulated Abca1; 2) induced lipidation of APOE4; and 3) reduced cognitive deficits, tau hyperphosphorylation and Aβ accumulation [125]. In a follow-up study using *APOE4* TR and *APOE3* TR mice, the authors showed that CS-6253 also normalizes plasma APOE4 lipidation and stability to match *APOE3* mice and additionally, this peptide was able to partially normalize plasma apoA-I and apoJ levels in *APOE4* TR mice [126]. Another strategy to upregulate Abca1 is by using ASOs against microRNA-33. Inhibition of microRNA-33 by ASOs in cultured neurons and *APP* transgenic mice reduces Aβ levels [127, 128]. With the assumption that these drugs do not disrupt lipidation state and the normal biological function of APOE3, these studies support the notion that activation of ABCA1 to stabilize lipidation profile of APOE4 is a viable therapeutic target. Taken together, these studies demonstrate that correcting the hypolipidation state of APOE4 may be enough to alleviate AD-type pathologies.

#### Small molecules as APOE4 structure correctors

The domain interaction property of APOE4 reduces its secretion from cells [129] and concurrently makes it protease-labile [130], leading to pathogenic effects [131]. Thus, another potential therapy would be the disruption of this APOE4 domain interaction using ‘structural correctors’ which are expected to negate the pathological consequences of this domain interaction (Table 1). A study using a FRET system coupled with high throughput screening identified several small molecules that could be used as structural correctors [132]. Treatment of Neuro-2a cells expressing APOE4 with such structure correctors caused the protein to become more ‘APOE3-like’ both structurally and functionally. By restoring mitochondrial cytochrome c oxidase levels, this treatment reversed some of the detrimental effects of APOE4 in Neuro-2a cells. In another study using a human cell line, similar effects were observed using the small molecule structure corrector PH002. The compound decreased APOE4 fragmentation, increased GABAergic neuron numbers, reduced phosphorylated tau and Aβ levels in a dose-dependent manner [55]. The studies provide proof of concept that disrupting the APOE4 domain interaction using structure correctors could be druggable target in AD.

#### APOE mimetic peptides regulate function via competing for receptor binding

Using peptide mimetics that are structurally similar to the lipid binding class A amphipathic helix found in apoE, it is possible to regulate the lipidation and secretion of APOE. These peptides are so designed as to promote cholesterol trafficking, anti-inflammatory signaling and anti-thrombotic effects – properties that have been used in targeting systemic disorders such as atherosclerosis and coronary artery disease [133] or acute brain injury models [134–136]. One example is an 18 amino acid peptide with no known natural homologs called 4F (Ac-D-W-F-K-A-F-Y-D-K-V-A-E-K-F-K-E-A-F-NH2) that binds to LDL (particularly oxidized phospholipids and unsaturated fatty acids) and HDL at a site that is recognized by APOE [137]. In primary glial cell cultures derived from humans or mice, 4F increased APOE lipidation and APOE secretion [137] and reversed aggregated Aβ-induced blockage of glial APOE secretion. In a second study using *APP* overexpressing Drosophila, two novel APOE mimetics, COG 112 and COG 113, prevented neurodegeneration and improved memory, though Aβ deposition was not changed [138]. This suggests that such APOE mimetics can alter AD-type dysfunction through altering lipid metabolism that may be independent of Aβ pathology. These peptides, when used in CVND-AD transgenic mice (SwDI-*APP*/*NOS2*(-/-)) improved memory as well as reduced Aβ plaques and phosphorylated tau levels [139]. One study showed that such mimetic peptides are efficacious in *APOE3 TR* or *APOE2 TR* mice, but had no effect in *APOE4 TR* mice [140], suggesting isoform-specificity. Another APOE mimetic peptide derived from the receptor binding region of APOE α helix, CN-105 (Ac-V-S-R-R-R-NH2) has successfully completed Phase I clinical trial in patients with intracerebral hemorrhage (ICH) (Table 1). This peptide is BBB penetrant and reduces neuroinflammation and neuronal injury in mouse models of acute brain injury mouse models [135, 136] but this peptide has not been tested in rodent AD models. Given the beneficial role of APOE mimetics, future studies in AD mouse models and cell culture systems with such mimetics are warranted.

#### Small molecule inhibitors of APOE-Aβ interactions

As previously stated, APOE, especially APOE4, is normally found within Aβ deposits [141]. Inhibitors of protein–protein interactions (PPI), once considered undruggable, are now emerging as a tour de force because of dramatic improvement in understanding of PPI scaffold chemistry [142]. An advantage of this method is that these are often naturally occurring molecules that can be very selective because of their precise targeting [143]. One such inhibitor that disrupts the binding of APOE to Aβ is a peptide mimetic called Aβ12-28P, which is a non-fibrillogenic and non-toxic Aβ derivative that happens to be BBB permeant [144]. This peptide, by blocking the binding of APOE and Aβ at residues 12 to 28, reduced Aβ-induced neurotoxicity in cell culture. Further studies revealed that Aβ12-28P had a strong pharmacological effect in vivo where systemic administration of the peptide resulted in reduction of Aβ deposits and in general a reduction of CNS Aβ in two different *APP* transgenic mouse lines [145]. Administration of Aβ12-28P also prevented working memory deficits in mice, bolstering its further translatability [145, 146].

APOE is expressed predominantly from astrocytes in the CNS [147, 148]. However, APOE synthesized by astrocytes can be neurotoxic, to the extent that specifically deleting astrocytic Apoe rescues spatial learning and memory deficits in the *APP*/*PS*1 mouse model [149]. This is also supported by a study that used a co-culture system of neurons and astrocytes to investigate the role of APOE on intraneuronal accumulation of Aβ [150]. Intraneuronal Aβ accumulation was higher in neurons co-cultured with wild-type mouse astrocytes compared to the cultures exposed to *Apoe* KO astrocytes reinforcing the idea that APOE plays a key role in Aβ proteinopathy. Treatment with Aβ12-28P, which disrupts APOE-Aβ interaction, significantly lowered the amount of intraneuronal Aβ as well as inhibited the loss of synaptic proteins in this co-culture system [150].

Another example of an inhibitor of APOE-Aβ interaction is the 6KApoEp peptide that inhibits the binding of APOE to the N-terminus of APP [151]. This peptide contains residues 133-152 of APOE protein conjugated to six lysine residues at the N terminus. When 6KApoEp was injected into the 5XFAD mouse model of amyloid pathology, both Aβ and tau pathologies were reduced concomitant with improved memory and hippocampal-dependent learning. However, 6KAPOEp therapy did not alter the cholesterol or APOE levels in 5xFAD mice. These results demonstrate that apoE-Aβ interaction inhibitors could potentially be used for therapeutic reduction of Aβ and tau burden in the CNS.

#### HDAC inhibition regulates endolysosomal function

Another group of researchers reported that HDAC regulates endolysosomal function [152, 153]. Initially, using yeast microarray databases, they identified Nhx1 as a major HDAC regulated factor induced during nutrient-limiting conditions [152]. Nhx1 is an endosomal Na+/H+ exchanger (eNHE) whose main function in yeast is vacuolar alkanization. The mammalian homolog of NHx1 was identified as Nhe6 that is regulated by cAMP-response element-binding protein (CREB) and plays a key role in nutrient- and HDAC-dependent regulation of endosomal pH [152]. This research group used three different pharmacological strategies to activate HDAC/CREB-dependent Nhe6 expression in immortalized astrocytes expressing APOE3 or APOE4 and observed that Creb-dependent Nhe6 expression corrected Aβ clearance deficits observed in *APOE4* astrocytes. In a second report, this research group could mechanistically relate this finding to dysfunction in LRP1 endocytosis [153]. Using both *Nhe6* deficient mice and immortalized *APOE4* astrocytes, they showed that *Nhe6* deficiency causes endosomes to become hyperacidic, which impedes Aβ clearance by impairing endocytosis of LRP1 [153]. Inhibition of HDAC could normalize Aβ clearance by restoring Nhe6 in the *APOE4* astrocytes. Though these HDAC inhibitors are efficacious in other systemic disorders such as heart failure [154] and cancer [155], the widespread clinical applications is limited due to selectivity issues and toxicity issues.

### Recalibrating APOE function using gene editing and gene therapy

Several experimental strategies have been tested to alter the prevalent apoE isoform in rodent models and human-derived induced pluripotent stem cells (iPScs) as a means to rectify the neurotoxic functions of APOE4. Various studies have used CRISPR-mediated or adeno-associated virus (AAV)-mediated gene delivery in these model systems. However, these strategies have to contend with ethical and safety hurdles before these can be translated to clinical settings.

#### CRISPR/Cas9 mediated gene editing

One promising method for gene editing is using the CRISPR (Clustered Regularly Interspaced Short Palindromic Repeats) system that has just entered Phase 1 trial for treatment of relapsed refractory multiple myeloma and related cancers (NCT03399448: University of Pennsylvania, Parker Institute for Cancer Immunotherapy, Tmunity Therapeutics). CRISPR/Cas9 basically functions like a pair of molecular scissors where an editable guide RNA leads the Cas9 ‘scissor’ to a specific site of the genome to cut where a different nucleotide sequence can then be inserted to correct a genetic defect [156]. CRISPR/Cas9 has already been proven successful in iPS cells, where cells derived from a healthy E3/E4 individual were converted into E2/E2, E3/E3, E4/E4, or an *APOE* KO genotype [157]. A second group used iPS cells derived neurons from an *APOE4* carrier and found that CRISPR-editing the *APOE4* reduced tau phosphorylation and inomycin-induced cell death [158]. Interestingly, though in the CNS APOE is mostly synthesized by astrocytes, this study showed that editing neuronal APOE to the E3 isoform in these iPS-derived neurons is sufficient to protect them from cytotoxic injury [158]. Another study generated different brain cell types and organoids from iPS cells derived from a human subject – on editing the *APOE4* allele to *APOE3* in these iPS-derived cells increased Aβ clearance and reduced Aβ in the organoid cultures [49]. This study shows that targeting APOE in various CNS cell types can lead to beneficial functional alterations in patient-derived in vitro systems. In animal models, CRISPR/Cas9 is relatively safe and has been successfully used to generate *APOE* KO in pigs and rats with little to no off-target incidents or mosaicism [159–161]. However, there is always the possibility of unexpected edits in the targeted and non-targeted portions of the genome leading to unanticipated side effects as well as triggering cancer risk [162, 163]. Inherent issues of CRISPR/Cas9 include off-target gene editing and mosaicism or where only some of the copies of the target gene are actually edited which could result in harmful side effects or unreliable treatment. Though data from iPS cells is extremely promising, much more research and ethical hurdles need to be cleared before gene editing with CRISPR/Cas9 is ready to be used as a clinical intervention.

#### AAV-APOE2 biologic therapy

*APOE4* has been established as the risk allele for AD, and *APOE2* is protective. This set the foundation for the idea that if APOE2 could replace or be overexpressed in *APOE4* carriers, there would be a compensatory beneficial therapeutic effect. Indeed, there is a current trial scheduled to start that intends to test the safety of AAV-APOE2 expression in *APOE4* carriers (Table 1). Patients will be infused with AAV-APOE2 in the cisterna magna and then followed up for at least 2 years to assess safety of this biologic therapy.

There is a robust rodent literature showing the effects of AAV-mediated APOE expression in primarily mouse models of amyloidosis. For example, intracerebral injection of AAV-APOE4 in *APP*/*PS1* and Tg2576 mice resulted in increased Aβ burden whereas AAV-APOE2 lowers Aβ burden [164]. However, a limiting factor of this study is that this was done in the presence of murine Apoe that may itself influence Aβ deposition. In a subsequent study from a second group, expression of AAV-APOE2 was shown to reduce Aβ plaque burden in a trigenic mouse (*APP*/*PS1*/*APOE4* TR) [165]. This study also showed that gene delivery of APOE2 was most effective before the onset of amyloid burden, suggesting that in order to be a successful therapy the AAV would need to be injected much before the onset of symptoms in patients which poses its own challenges. Further translational studies on non-human primates revealed that intra-cisternal delivery of AAV-APOE2 led to widespread expression in the CNS which established a safe procedure for CNS delivery of biologics [166]. Because of the inherent risk in any surgical procedure inside the CNS, whether such AAV-APOE2 biologics can be directly delivered into the AD-affected areas of the human CNS needs to be cautiously determined. However as Zhao et al showed in mice, even intrathalamic injections were modestly effective in reducing Aβ burden in neuroanatomically distant areas such as hippocampus [165]. Another confounding variable is that while APOE2 may decrease Aβ plaque formation, it may increase tau phosphorylation [12]. By injecting AAV-P301L tau into *APOE* TR mice, this research group found that mice expressing APOE2 had higher NFT levels compared to mice expressing APOE3 or APOE4. Along with data showing genetic association between *APOE2* with PSP in humans, this brings up the question if overexpression of APOE2 could inadvertently exacerbate tau pathology while alleviating amyloid burden. In addition, questions regarding effective dosage to achieve optimum biodistribution and cell type transduction, pre-existing host immunity and long term CNS consequences still remain safety concerns in AAV therapies. In addition, while AAV-APOE2 gene therapy has promise, more knowledge on the neuropsychological and neuropathologic consequences of APOE2 overexpression is needed.

### Lifestyle and diet can also regulate APOE function

Metabolic syndrome (MetS) can be characterized as a cluster of disorders that are associated with atherosclerosis, diabetes, hypertension, and has been linked to dementia in general. A few studies have indicated that the *APOE4* allele is associated with increased risk of MetS leading to dementia [167]. Multiple case studies have thus examined the relationship of interventions in lifestyles such as, but not limited to, diet and exercise to reduce the risks associated with the *APOE4* isoform. Though there is still no strong precedent for these lifestyle factors to effectively reduce metabolic dysfunction and AD risk via affecting APOE function, these interventions hold promise as future and easily translatable strategies in the personalized medicine niche due to their safety profiles.

#### Exercise

Based on epidemiological records and rodent studies, an intuitive therapeutic strategy for AD patients is exercise. Exercise increases cerebral blood flow, neurogenesis, and hippocampal volume as well has a positive impact on memory in humans [168, 169]. In wild type mice, exercise resulted in prevention of age-related neurovascular changes, especially in the context of the Apoe gene [170]. This was consistent with the idea that APOE plays a key role in functional impairment of the neurovascular unit during aging and exercise can reverse these effects by modulating neurovascular health.

Physical exercise can have a beneficial effect in AD type dementias by altering neuroplasticity as observed in both human case studies and rodent studies [171, 172]. However, there are still unresolved issues regarding the relative efficacies of different exercise regimens and presence of sex-dependent effects [173]. In a cohort of 201 cognitively normal adults, *APOE4* carriers who do not exercise frequently were shown to have an increased risk of Aβ deposition [174]. However, this study did not report how many of these sedentary *APOE4* carriers went on to develop actual AD-type dementia. A more recent study with 200 individuals diagnosed with mild AD addressed this issue, examining if exercise held any cognitive or physical improvements for *APOE4* carriers [175]. The data did support that exercise intervention improved cognitive function, and was found to be more beneficial to *APOE4* carriers. However, out of the five tests for cognition, only one test showed a statistically relevant correlation between exercise and *APOE4* status. Along with the small sample number and lack of information on the ethnicities of the cohort, larger studies would be required to validate any of the conclusions and extend its application in the clinical setting.

#### Statins

Statins, or HMG-CoA reductase inhibitors, are a class of drugs that are typically prescribed to lower cholesterol levels in the blood. Researchers have postulated that increased brain cholesterol levels, or at the very least disruption of lipid homeostasis, influences AD pathology and risk. Epidemiological studies support that higher serum cholesterol levels are linked to increased risk of AD independent of *APOE* genotype [176–178]. A series of epidemiological studies looked at the effect of statins on dementia in general, spurred by observations that usage of statin led to a significantly lower rate of cognitive decline over 6 months [179]. However, more recently the LEADe trial of 2010 and the CLASP study of 2011 that assessed the use of statins in AD patients found no net benefit or harm in terms of cognitive decline relative to the placebo group [180, 181]. Further support for the idea that statins do not generally benefit AD patients comes from another systematic review [182]. These findings, however, contradict another large study of Medicare beneficiaries which established a beneficial association between statin use and reduced AD incidence in specific populations [183]. The data, however, showed wide variations in efficacy of statins based on race and sex; for example, pravastatin was associated with reduced AD risk only among white women whereas atorvastatin was efficacious in white women, black women and Hispanic men. This finding suggests that overall statin use may not be beneficial for all people at risk for AD, but in a future of personalized medicine, physicians should consider whether statins could have higher health impact in specific patient populations based on sex, ethnicity, prevalent health conditions, and *APOE* genotype.

#### Ketogenic diet

Modern diets that are high in carbohydrates and low in fats elevate blood glucose levels after ingestion and can alter APOE function through glycation and oxidative damage [184]. These diets are associated with impaired brain glucose metabolism, which is an AD biomarker. Feeding *APOE TR* rodents a high fat diet affected the plasma levels (E4>E3) and hippocampal levels (E3<E4) of APOE in an isoform-dependent manner [185]. Given that *APOE4* carriers have abnormally low rates of glucose metabolism compared to other *APOE* genotypes, it is possible that these diets may profoundly alter metabolic status in *APOE4* patients [186]. A proposed method to supplement brain health could be the use of ketone bodies that are produced by using a ketogenic diet, or a high-fat low-carbohydrate diet (reviewed in [187]) that can alter the microbiome and improve neurovascular functions in young healthy mice [188]. In a small clinical study on AD patients with mild cognitive impairment (NCT02984540), certain gut bacteria showed significant correlation with AD CSF biomarkers (Aβ and phosphorylated tau) and further a modified Mediterranean-ketogenic diet altered gut bacterial profile [189], suggesting that such diets can regulate AD biomarkers through regulating gut microbiome and associated metabolites. Some diet intervention trials have reported that such regulated diets might have an effect on the neuropsychiatric profile of early AD patients [190], and some interventions show an APOE-dependent effect [191, 192]. These studies, however promising, need to be considered as they are – isolated case studies or small trials that require larger placebo controlled investigation for validation and further studies in rodent models are warranted.

#### Insulin resistance and APOE

As previously stated, diabetes and impaired insulin signaling are factors that increase the risk for MetS and are associated with increased AD risk [193, 194]. Peripheral insulin resistance is associated with lower cerebral glucose metabolism, which is also generally true of *APOE4* carriers, and this is further associated with poorer memory performance [195]. However, a clinical trial using insulin nasal sprays showed a complicated sex/*APOE* interaction. In the *APOE4* negative group, male AD patients improved in cognitive function whereas women worsened, whereas in the *APOE4* group, both sexes remained equally stable [196, 197].

In mice, the role of Apoe in insulin signaling was established in a study that showed that deletion of Lrp1, a major Apoe receptor, led to impaired brain insulin signaling and glucose metabolism [198]. Studies in *APOE4* TR mice showed that age along with peripheral insulin resistance contribute to the insulin signaling impairment in the brain by trapping the insulin receptor inside endosomes and contributing to impaired glycolysis [60]. With the current emerging knowledge on the regulation and function of brain insulin signaling, there is a need for further research into how insulin/glucose metabolism intersects with dementia in APOE isoform-dependent manner.

### Neuroinflammation and cerebrovascular integrity in the context of APOE function

Evidence suggests that inflammation as well as cerebrovascular damage play a crucial role in the pathogenesis of AD. APOE has been shown to predispose carriers to different neuroinflammatory profiles depending on the isoform. For example, in the ROS/MAP kindred of LOAD, the protective role of the *APOE2* haplotype could be traced to its counteracting a pathologic microglial signature [199] though *APOE4* did not show a corresponding pathologic effect on aged microglia [199, 200]. In mouse models, both *Apoe* KO mice and *APOE4* TR mice upregulate pro-inflammatory phenotype when challenged with bacterial lipopolysaccharide [201]. A recent paper suggested that mouse Apoe and human APOE4 act as a direct checkpoint inhibitor by binding to the complement C1q and attenuating the classical complement cascade [202]. This work has spurred interest to not only investigate the function of glia-specific APOE in the CNS but how this impacts the neurovascular unit including the BBB. This line of research has not yet identified any druggable candidates but future research into neuroinflammation and peripheral inflammation may yield potential targets that can be targeted in an *APOE* genotype-dependent manner.

#### TREM2

Microglia are resident immune cells in the brain that help maintain CNS homeostasis and can initiate inflammatory reactions when this homeostasis is perturbed. In AD, microglia can become chronically dysfunctional [203]. Recent genome wide association studies have identified several microglial genes that regulate AD risk, foremost among them being TREM2 [204]. The current state of thought is that variants of TREM2 that increase AD risk are loss of function mutations [205–207]. Recent studies have implicated a close relationship between TREM2 and APOE. APOE has been found to regulate the function of a subset of microglia, which under the control of TREM2 can adopt a damage associated microglia (DAM) phenotype [208] that is analogous to a toxic molecular signature of disease-associated microglia (MGnD) observed in several animal models including AD model [209]. This APOE-dependent phenotype is induced in phagocytic microglia in the presence of apoptotic neurons and activation of the TREM2-APOE signaling pathway results in functional impairment of the microglia. The authors suggested that the switch from homeostatic to neurodegenerative state in AD-associated microglia is an initial response to neuronal injury compounded by a failure to switch back to a functional state. Several follow-up studies have now shown that Aβ is also a ligand of Trem2 [210, 211], implicating the TREM2-APOE pathway directly in AD pathogenesis. A recent report showed that loss of Trem2 accelerates amyloidogenesis in mice by reducing microglial function but these newly seeded deposits show reduced amounts of Apoe compared to mice carrying Trem2 [212]. Together this data suggests that microglia, through Trem2 mediated signaling, can regulate apoE co-deposition around Aβ deposits, which further has significance in terms of Aβ clearance based on specific APOE isoform [164]. Independently, in a mouse model of tauopathy-mediated neurodegeneration, reducing microglial activity through pharmacological methods increases soluble APOE, reduces tauopathy and rescues neurodegeneration in *APOE4* mice [54]. This report did not specifically look at Trem2 though another previous report had ahown that attenuating microglial Trem2 is protective against tau-mediated neurodegeneration [213]. Given that this scenario of tripartite interactions between Aβ, tau and APOE is mediated through microglial homeostasis, it is tempting to suggest that targeting microglial TREM2 functions can result in *APOE*-isoform dependent therapeutic benefits. Of note, a recent report showed that the ectodomain form of TREM2, soluble TREM2, is protective in an amyloid mouse model by enhancing microglial metabolism of Aβ [214] and triggering microglia to an active state [215]. Given that TREM2 facilitates microglial degradation of Aβ preferentially complexed with LDL [210], this raises the intriguing possibility that soluble TREM2 may have therapeutic promise. However, another cell culture study seemed to indicate that AD-associated TREM2 risk variants do not show altered binding affinity for Aβ or APOE [211], raising the conundrum regarding whether TREM2-Aβ interaction is functionally dependent on specific *APOE* genotype.

#### Blood Brain Barrier

The BBB is composed of a layer of tightly packed endothelial cells which keeps out neurotoxins and pathogens from the brain, imparting a sort of unique ‘immune privileged’ milieu during healthy conditions. In AD, BBB dysfunction and leakiness precedes neurodegenerative changes, brain atrophy, and dementia [216]. This finding has encouraged researchers to look into how BBB breakdown relates to neurodegeneration in a series of AD mouse models including *APOE* models. It has been suggested that APOE is essential for maintaining BBB integrity as the BBB is leaky in *Apoe* KO models [35, 36, 217]. Further, APOE triggers BBB breakdown in an isoform-dependent manner in an in vitro model (E4>E3) [91], though another group reported that the BBB is largely intact at least in young *APOE4* TR mice [40]. This raises the possibility that APOE4 mediated BBB disruptions can be localized to selectively vulnerable brain regions or may depend on other factors, such as aging or presence of amyloid angiopathy. Another group of researchers found that *APOE4* mice had higher levels of the cyclophilin A (CypA)-matrix metalloproteinase 9 (MMP-9) in the pericytes. Since pericytes make up the BBB, this can lead to degradation of tight junctions and basement membranes and leakiness of the BBB [36]. Other studies using radioactive tracers in mouse models or using in vitro model of mouse brain microcapillaries showed that APOE3 and APOE2 mediate Aβ clearance through a faster route via LRP1 across the BBB while APOE4 mediates Aβ clearance through VLDR at a much slower rate possibly contributing to CNS accumulation of Aβ [43]. These studies revealed a potential therapeutic target, where researchers genetically and pharmacologically inhibited the CypA-MMP-9 pathway which resulted in repairing the BBB and reversing the neurodegeneration [36]. Curiously, loss in BBB integrity would also imply that drugs (such as antibodies) administered peripherally could gain easier access into the brains of *APOE4* individuals, leading to higher bioavailability. On the whole, more studies are still required to establish the relationship between *APOE* genotype and BBB integrity and how this is altered in the context of neurodegenerative dementia of the elderly.

### Critical Challenges for targeting CNS resident APOE

One of the most critical challenges for any AD therapeutic is optimizing the route and mode of administration so as to achieve effective bioavailability by bridging the BBB. A major area of research is now devoted to discovering cutting edge technologies that can safely breach the BBB. One option is to use the so-called Trojan horse strategy utilizing bifunctional molecules, one arm of which can be used to shuttle the APOE therapeutic across the BBB as has been demonstrated for anti-Aβ antibodies [218]. Another new technique is using pulsed ultrasounds that would create transient openings in the BBB allowing the APOE therapeutic to reach the substrate, as has been done to optimize chemotherapy in glioblastoma patients [219]. Perhaps another alternative would be to use gene therapy vectors to deliver a beneficial (E2) or even the neutral form (E3) of *APOE,* using specific AAV capsid serotypes that are preferentially neurotropic even when administered in the periphery [220, 221]. Each one of these tools have their shortcomings – for example, the bridging molecules used for the Trojan horse strategy are not particularly specific for the BBB, leading to potential dilution or even unwanted peripheral side effects. Likewise, the pulsed ultrasound and AAV based approaches have unknown long-term health implications. Interestingly, a 20 amino acid stretch of the APOE protein itself has been successfully utilized for shuttling therapeutics across the BBB in a mouse model of lipofuscinosis, a pediatric neurodegenerative disorder [222, 223], suggesting the possibility of using endogenous shuttling signals for efficacious delivery through the BBB. Even with these exciting breakthroughs, several challenges remain: if administered peripherally, how do we prevent the APOE therapeutic to be titered by the peripheral pools of APOE, or worse, cause systemic metabolic dysfunction and additionally, how to safely guide the therapeutic to the affected brain regions or cell types once inside the brain.

## Targeting APOE in other dementias

Aside from its established role in AD, not much is known about how APOE influences disease pathogenesis in AD related dementias such as Fronto-temporal dementias (FTD), dementia with Lewy bodies (DLB) and vascular dementia. Consequently, very few mechanistic and therapeutic studies in mouse models are available.

The APOE2 allele is associated with an increased risk of ALS-FTD [224]. In another study, APOE2 and APOE4 alleles showed protective and increased disease risk effects, respectively, for FTD subtypes such as behavioral variant FTD and semantic dementia, though potential overlaps between clinical diagnosis of FTD and AD cannot be completely ruled out in this study [225]. Similarly, APOE4 appears to be a risk factor for DLB [226] and vascular dementia [227]. There is no direct association of APOE with other atypical parkinsonism syndromes with dementia such as corticobasal degeneration (CBD), multiple system atrophy (MSA) and progressive supranuclear palsy (PSP) [228]. Knocking out mouse *Apoe* resulted in delayed neurodegeneration in a mouse model of synucleinopathy [229]. In mouse studies, both APOE4 as well as APOE2 increased tauopathy burden in two different mouse models [11, 12], raising intriguing possibilities of how APOE might interact with tau in the presence of co-morbidities (such as Aβ and α-synuclein).

## Conclusions

**Fig. 2:**
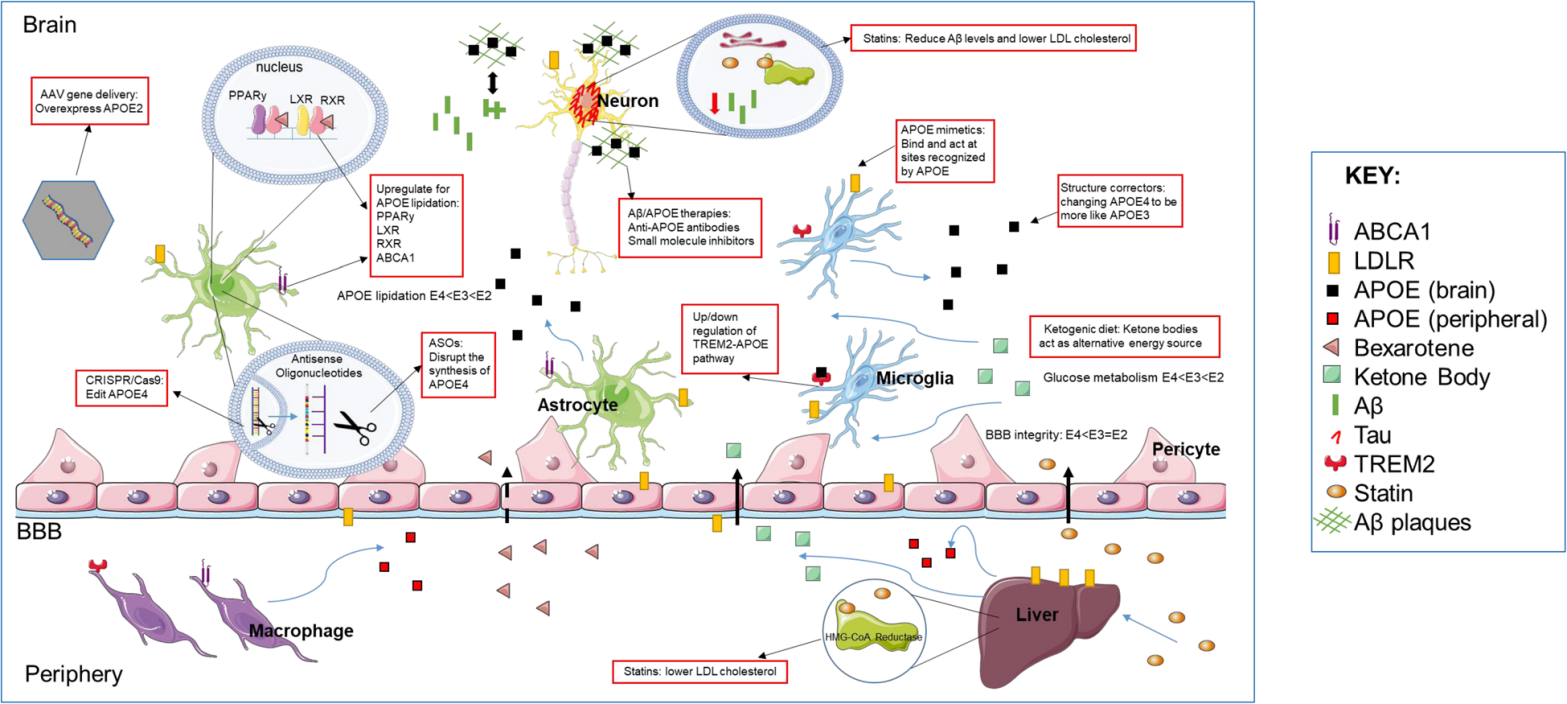
A schematic to illustrate the various targets for APOE-based AD therapeutics**.** See the main text for details. Black arrows pointing to the red text boxes indicate a mode of intervention while blue arrows indicate the movement of the drug or protein. Black solid arrows crossing the BBB show permeability while black dashed arrows show BBB semi-permeability. ABCA1: ATP-binding cassette transporter A1; LDLR: low density lipoprotein receptor; APOE: apolipoprotein E; Aβ: amyloid-β; TREM2: triggering receptor expressed on myeloid cells 2

APOE not only impacts lipid metabolism but various CNS functions and neurodegenerative proteinopathy in AD in an isoform-dependent manner. The current evidence highlights how APOE isoform determines physiological homeostasis in the brain and how several APOE-targeted therapeutic approaches can have corrective or preventive outcome(s) in neurodegenerative proteinopathies, particularly in AD (Fig. 2). Many of these experimental approaches are validated in various cellular or animal models, with the overall perception that current APOE-targeted therapies would be more effective at prevention rather than treatment of those already in the throes of the neurodegenerative cascade. If or when these treatments make it through clinical trials, the potential benefit could be greatest for *APOE4* carriers, where early intervention would slow the rate of decline (neuropathologic or neuropsychiatric) though it is unlikely to entirely stop the progression of disease. This is exemplified in some of the rodent amyloid models, where early intervention led to reduction of Aβ deposits but not complete clearance. However, if future research shows that APOE alters other AD related proteinopathies in these patients, such as tau or α-synuclein or inflammation either directly or through altering Aβ levels, then certain APOE directed therapies may have more profound multi-target effects in an APOE isoform-dependent manner. Additionally, APOE4 targeted therapies might also become adjuvants to other multimodal treatments that would target the more age-advanced pathologies, such as neuroinflammation or BBB leakiness [230]. Advancements in biomarkers for earlier diagnosis and prognosis of AD, especially in an APOE-informed population, would be invaluable for targeted therapies in an emerging era of precision medicine. Additionally, how such interventions will alter peripheral lipid homeostasis and vascular function would also need to be determined. The safety profile of any therapeutic will thus need to balance the total amount of APOE, lipidation profile of APOE, vascular risk factors, inflammatory phenotype and systemic effects. With this taken together, APOE-targeted therapeutic strategies remain a propitious area of research for preventing or delaying the onset of AD type dementias.

## Data Availability

Not applicable.

## Abbreviations

AAV: Adeno-associated virus
AD: Alzheimer’s disease
ALS: Amyotrophic Lateral Sclerosis
APOE: Generic apolipoprotein E including human form
Apoe: Mouse apolipoprotein E
APP: Amyloid precursor protein
ASO: Antisense oligonucleotides
Aβ: Amyloid β
BBB: Blood brain barrier
CBD: Corticobasal degeneration
CETP: Cholesteryl ester transfer protein
CNS: Central Nervous System
CREB: cAMP-response element-binding protein
CRISPR: Clustered Regularly Interspaced Short Palindromic Repeats
CypA: Cyclophilin A
DAM: Damage associated microglia
DLB: Dementia with Lewy bodies
EGCG: Epigallocatechin gallate
eNHE: Endosomal Na+/H+ exchanger
FTD: Fronto-temporal dementias
HDAC: Histone deacetylase
HDL: High Density Lipoprotein
ICH: Intracerebral hemorrhage
iPSC: Induced pluripotent stem cell
LDL: Low-density lipoprotein
LOAD: Late onset AD
LXR: Liver X receptors
MetS: Metabolic syndrome
MMP-9: Matrix metalloproteinase 9
MSA: Multiple system atrophy
NFT: Neurofibrillary tangle
PNS: Peripheral nervous system
PPARγ: Peroxisome proliferator-activated receptor γ
PPI: Protein–protein interactions
PS1: Presenilin-1
PS2: Presenilin-2
PSP: Progressive Supranuclear Palsy
RXR: Retinoid X receptor
sAD: Sporadic AD
SILK: Stable isotope leveling kinetics
TR: Targeted replacement
TREM2: Triggering receptor expressed on myeloid cells 2
VLDL: Very low density lipoprotein
